# Supplementary material for: Public perception of an important urban estuary: Values, attitudes, and policy support in the Biscayne Bay-Miami Social Ecological System
Source: PLoS One. 2023 Oct 10;18(10):e0287930. doi: 10.1371/journal.pone.0287930 (PMC10564173; doi:10.1371/journal.pone.0287930)
Supplement: Attachment — Submitted filename: Response to reviewers.docx [file pone.0287930.s001.docx]

**Response to reviewers**

***I thank the reviewers and editor for their support and helpful comments on this manuscript. I have carefully reviewed the manuscript and incorporated suggested changes. Point-by-point responses are included in-line below in bold.***

***Kind regards,***

***Julia Wester***

1. Please ensure that your manuscript meets PLOS ONE's style requirements, including those for file naming. The PLOS ONE style templates can be found at

https://journals.plos.org/plosone/s/file?id=wjVg/PLOSOne_formatting_sample_main_body.pdf and

https://journals.plos.org/plosone/s/file?id=ba62/PLOSOne_formatting_sample_title_authors_affiliations.pdf

**The manuscript has been reviewed for formatting requirements.**

2. Please provide additional details regarding participant consent. In the ethics statement in the Methods and online submission information, please ensure that you have specified (1) whether consent was informed and (2) what type you obtained (for instance, written or verbal, and if verbal, how it was documented and witnessed). If your study included minors, state whether you obtained consent from parents or guardians. If the need for consent was waived by the ethics committee, please include this information.

If you are reporting a retrospective study of medical records or archived samples, please ensure that you have discussed whether all data were fully anonymized before you accessed them and/or whether the IRB or ethics committee waived the requirement for informed consent. If patients provided informed written consent to have data from their medical records used in research, please include this information.

**The following details have been added to the methods: “At the start of the survey participants were presented with information about the study and provided written consent in the form of checking a box to proceed before they were able to begin the survey.”**

3. We note that you have stated that you will provide repository information for your data at acceptance. Should your manuscript be accepted for publication, we will hold it until you provide the relevant accession numbers or DOIs necessary to access your data. If you wish to make changes to your Data Availability statement, please describe these changes in your cover letter and we will update your Data Availability statement to reflect the information you provide.

**Data has been made available via Dryad at the following location:** [**https://doi.org/10.5061/dryad.1rn8pk106**](https://doi.org/10.5061/dryad.1rn8pk106)

4. Please review your reference list to ensure that it is complete and correct. If you have cited papers that have been retracted, please include the rationale for doing so in the manuscript text, or remove these references and replace them with relevant current references. Any changes to the reference list should be mentioned in the rebuttal letter that accompanies your revised manuscript. If you need to cite a retracted article, indicate the article’s retracted status in the References list and also include a citation and full reference for the retraction notice.

**No changes have been made to the reference section. No retracted papers have been cited.**

Author are advised to focus on language and grammatical and text rearrangement for making a continuous flow for easy reading

**I have reread and lightly edited the manuscript for flow and clarity.**

Reviewer #1: Abstract:
1. Re-write abstract- with brief introduction, importance of study, brief methodologies adopted, salient achievements and conclusion.

**I thank the reviewer for their support of the manuscript and helpful comments here. I have revisited the abstract and believe that each of the components listed have been included already.**

Conclusion:
1. Suggested that author added references in manuscript conclusion which is not required to add references in conclusion. I suggested that re-write the conclusion in brief-way with suggestions.

**References have been removed from the conclusions.**

Reviewer #2: The study on Biscayne Bay shows the multiple parameters including the ecological interaction as well as physical interaction towards the water body by the public. It can be use for multiple purposes if the management practices adopted in a sustainable manners. Some where coma (,) is use with the word and like as (, and) so please correct it.

**We thank the reviewer for their support of the manuscript. I have reread and lightly edited the manuscript for grammatical or syntactical errors.**
